# Diagnostic accuracy of intraoperative CT-imaging in complex articular fractures – a cadaveric study

**DOI:** 10.1038/s41598-020-61267-w

**Published:** 2020-03-11

**Authors:** M. Luxenhofer, N. Beisemann, M. Schnetzke, S. Y. Vetter, P. A. Grützner, J. Franke, H. Keil

**Affiliations:** 1 MINTOS research group – Medical Imaging and Navigation in Trauma and Orthopedic Surgery, Department for Trauma and Orthopedic Surgery, BG Trauma Center Ludwigshafen at Heidelberg University Hospital, Ludwig-Guttmann-Str. 13, 67071 Ludwigshafen, Germany; 2 AGiTEC – Working Group for intraoperative imaging and integration of technologies of the DGOU, Berlin, Germany

**Keywords:** Trauma, Orthopaedics, Experimental models of disease

## Abstract

Anatomic reconstruction of articular fractures is one of the critical factors in later achieving good functional outcome. Intraoperative 3D imaging has been shown to offer better evaluation and therefore can significantly improve the results. The purpose of this study was to assess the difference between intraoperative three-dimensional fluoroscopy (3D) and intraoperative computed tomography (iCT) imaging regarding fracture reduction, implant placement and articular impressions in a distal humeral fracture model. AO type 13-B2 fracture pattern were created in upper extremity cadaver specimens. Articular step-offs, intra-articular screw placement and intraarticular impressions of different degrees of severity were created. All specimens had imaging performed. For each articular pattern 3D fluoroscopy in standard (3Ds) and high quality (3Dh) were performed (Arcadis Orbic, Siemens, Germany) as well as an intraoperative CT scan (iCT, Airo, Brainlab, Germany). Three observers evaluated all imaging studies regarding subjective and objective parameters. iCT is more precise than 3D fluoroscopic imaging for detection of articular impressions. Articular step-offs and intraarticular screw placement are similar for iCT and 3D. Subjective imaging quality is the highest for iCT and lowest for 3Ds. Intraoperative CT may be particularly useful in assessing articular impressions and providing a good subjective image quality for the surgeon.

## Introduction

### Distal humeral fractures

Fractures of the distal humerus are rather rare in adults, they comprise approximately 2% of all fractures overall and one-third of all humeral fractures^1,2^. The incidence of distal humerus fractures in adults is 5–6/100.000/year^3^ with the average age being 48.4 years^1^. Distal humerus fractures are often results of high-energy trauma in young, active, male patients (motor vehicle accidents and sporting activities) and low-energy injuries in elder women^3–5^. Fractures of the distal humerus with their complex anatomical structures, high biomechanical load applied to the implants as well as associated soft tissue damage pose difficult challenges for the surgeon^3^. Fractures of the distal humerus, some of which are treated with multiple osteosynthesis plates, are therefore a good example for testing the limits of intraoperative imaging.

### 3D-Imaging

As two-dimensional imaging is a projection imaging technique and thus shows limited information, it might lead to misjudgment of the fracture site and surgical result^6^. Complex anatomical regions such as spine, pelvis or articular surfaces pose a particular high level of complexity and demand precise judgment of the fracture site to ensure successful osteosynthesis^7^. Various studies have shown that CT imaging provides significantly better diagnostic results than 2D imaging^8–11^. Wicky *et al*. have shown that in over 50% surgical planning has been altered after preoperative 3D-reconstruction^12,13^. The advantages of preoperative diagnostic 3D-imaging also apply to intraoperative 3D-imaging - although the setting of intraoperative imaging is different and more complex.

### Intraoperative 3D-Imaging

Intraoperative fluoroscopy has become the standard method for assessing reconstruction of joint surfaces^14,15^. Still, in distinct situations, 2D-imaging may not be sufficient for the evaluation of fracture reduction and implant placement particularly in complex anatomical regions^16^. Several studies have shown that intraoperative 3D-imaging with a special C-arm offers significantly superior diagnostic results compared to 2D-imaging^8–11,17–19^. Fluoroscopic 3D-imaging with a rotating C-arm is also called cone beam computed tomography (CBCT). Traditional CT uses fan-shaped X-ray beams for scans while cone-beam computed tomography uses divergent X-rays forming a cone-shaped beam. For differentiation between the two imaging modalities fluoroscopy generated scans with a C-arm will be called “intraoperative 3D-imaging” and CT-scans generated by a mobile traditional (fan beam) CT-scanner “intraoperative CT (iCT)”. 11–41% of fracture reductions which have been deemed sufficient in the surgeon’s opinion after routine 2D imaging have been revised after intraoperative 3D-imaging^14,20,21^. Intraoperative 3D-imaging improves patient care and outcome through improved evaluation of fracture reduction and implant placement, especially in complex anatomical regions^16,22,23^ and therefore became an established tool in trauma surgery^24^. 3D-imaging appears especially helpful in operative situations with limited direct sight of the non-planar articular surface (e.g. distal humerus fractures AO type B and C) since it offers additional information^24^. Still, intraoperative fluoroscopic 3D-imaging with a mobile C-arm also has its limits: Overweight and unfavorable positioning of the patient partially limit free rotation and thus imaging by the motorized C-arm^13^. Restrictions also exist due to the relatively reduced data set^13^. Intraoperative detection of insufficient fracture reduction or implant placement allows immediate correction of the malposition and can thus prevent revision operations. The additional effort of OR time has been determined to be about 7.5 min with fluoroscopic imaging for fixation of intraarticular fractures^20^ or respectively 18–34 min using intraoperative CT Airo for spinal navigation – with rapid improvement of surgery times after few procedures^25^.

Intraoperative CT-imaging allows multiplanar reconstruction of the fracture site with a higher imaging quality and bigger field of view compared to 2D and fluoroscopic 3D-imaging^26^. Disadvantages are the comparatively high radiation exposure - although the radiation exposure for the surgeon is lower with iCT than with 2D-fluoroscopy as the staff leaves the room during acquisition - as well as the high investment and operating costs^26^. However, Sanborn *et al*. found that the iCT had the lowest cost and comparable effectiveness as a 2D intraoperative imaging paired with postoperative CT scanning for confirming pedicle screw placement^27^. A number of studies are assessing the usability of the intraoperative CT Scanner Brainlab Airo for posterior instrumentation of the thoracolumbar spine^25^. They show a good usability and precision but have not rated imaging quality.

The intraoperative evaluation with iCT may benefit from the higher resolution of bone structures as well as the larger field of view compared to 3D‐fluoroscopy^26^. The objective of the current study was to evaluate the advantages of intraoperative CT imaging in assessing fracture reduction and implant placement compared to intraoperative fluoroscopic 3D-imaging with a mobile C-arm.

## Methods

All experimental protocols were approved by the local authorities Ethics Commission (*Ethics Committee of the Medical Association of Rheinland-Pfalz*, Reference No. 837.296.17 (11130)) and performed in accordance with their guidelines and regulations. The used specimens were provided in cooperation with the *Institute for Macroscopic and Clinical Anatomy* of the University of Graz. The body donors consented to the donation of their bodies for research purposes during their lifetime.

### Specimen preparation

**Figure 1:**
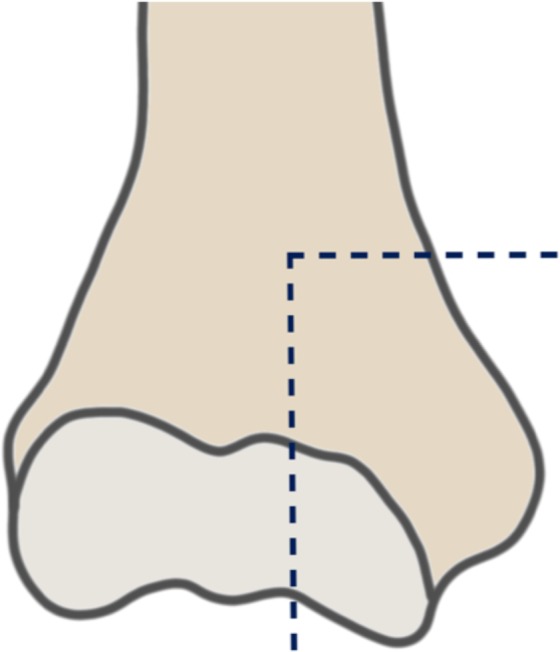
Osteotomy line of the distal humerus creating a medial fragment.

Ten upper extremity cadaver specimens were used for the study. A posterior approach via osteotomy of the olecranon was performed. An oscillating saw was used to create an osteotomy of the distal humerus, resulting in an AO Type B2 humeral fracture (see Fig. 1), as well as an osteotomy of the olecranon.

**Figure 2:**
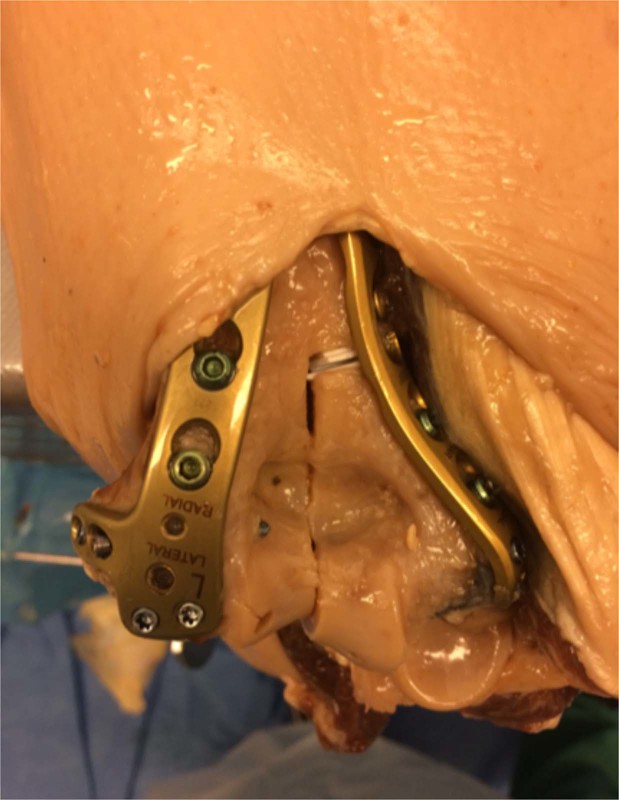
Osteosynthesis of a left humerus specimen with double plating.

The medial fragment was than reattached by double plating with two plates perpendicular to each other in AO standard technique (VA LCP distal humerus 2.4/3.5 mm, Synthes, Germany) (see Fig. 2). Plating of the olecranon was also performed (Synthes LCP Olecranon plate, Synthes, Germany). After performing imaging, the osteosynthesis material was then loosened, and articular step-offs of the humerus fracture of 1 mm to 4 mm in 1 mm increments were created in succession. Different patterns of screw placements and articular impression were established for each articular setting.

### Radiographic analysis

All specimens had imaging performed in five settings each creating 50 articular patterns.

For each articular pattern 3D fluoroscopy with a clinical standard 3D-imaging C-Arm (Arcadis Orbic, Siemens, Germany) in standard (3Ds, 50 images/scan) and high resolution (3Dh, 100 images/scan) were performed as well as an intraoperative CT scan (iCT, Airo, Brainlab, Germany). The scan parameters used are stated in Table 1. These parameters are the standard pre-set clinical settings of the devices, given by the manufacturer in regard to the anatomical region. The field-of-view (FOV) is fixed in 3D fluoroscopy. Technically, a cylindric volume is generated that is smaller than the detector. Due to the generic post-acquisition processing, depending on the actual device used, the actual visualized volume is smaller. In case of the device used in this study, the reconstructed 3D volume is a cubic volume with an edge length of 15 cm. The FOV in iCT was set to a cylindric volume of 37 × 10 cm with the center of the fossa in the isocenter of the device. The iCT has two standard FOV diameters, with 37 cm being the smaller setting. To allow standard conditions, this setting was not further changed.

**Table 1:** Scan parameters for each modality.

	DAP [mGycm^2^]	kV	DLP [mGycm]	Recon Volume [cm^3^]	Voxel size [mm]^3^
iCT		120	89,01	10752	1
3Ds	37.9	62.43		1728	0.475
3Dh	75.7	62.29		1728	0.475

Three independent researchers (medical student, surgical resident and experienced surgical attending) randomly evaluated all of the performed scans. Evaluation was done with Horos Medical image viewer. As in clinical routine, the observers were free to adjust the levels, contrast, brightness and zoom of all scans to ensure best subjective assessability for each parameter. As both imaging modalities use isotropic voxels, orientation of the planes was expected not to impair the assessability of the pathologies.

Imaging was evaluated for the following parameters:

- image quality as defined by the assessability score.
- articular step-off measured in maximal expression [mm].
- articular impressions measured in maximal expression [mm].
- intraarticular screw placement measured in maximal expression [mm].

The subjective image quality was assessed using the assessability score (see Table 2)^21^:

**Table 2:** Assessability Score.

0	<1/3 of the articular surface visible
1	>1/3 of the articular surface visible
2	No limitations in visibility of articular surface

### Statistical analysis

Statistical analysis was performed in cooperation with the *Institute of Biometrics, University of Heidelberg*. Binary scaled data (e.g. correctly analyzed scan [yes/no]) were analyzed using Cochran’s *Q* test followed by an exact McNemar’s post hoc test. Ordinally scaled variables (e.g. assessability score) were analyzed with the help of Friedmann-Test. For post hoc analysis Wilcoxon signed-rank tests was conducted with a Bonferroni correction applied, resulting in a significance level set at *p* < 0.017. Interval or ratio scaled variables were evaluated using a repeated measures ANOVA and Bonferroni-adjusted post hoc analysis. The interrater agreement was statistically analyzed using Cohen’s kappa.

## Results

In order to compensate for the differences between observers, the median of the evaluations was calculated for each parameter. All further calculations were based on the median values of the various observers.

### Assessability score

**Figure 3:**
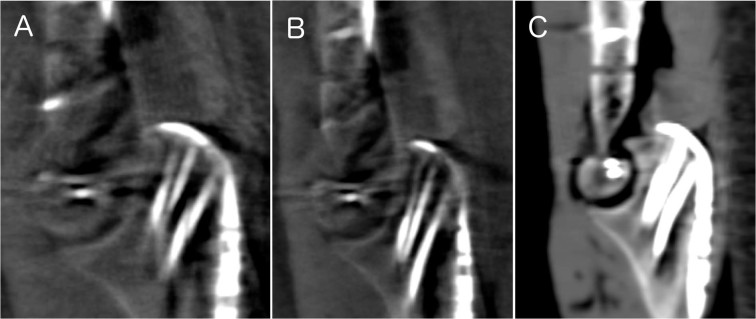
Assessability of the different imaging modalities (**A**: 3Ds, **B**: 3Dh, **C**: iCT).

The mean assessability score was 0.60 [n = 50] in 3Ds, 0.96 [n = 50] in 3Dh and 1.80 [n = 50] in iCT. There was a statistically significant difference in assessability score between all scan modalities, *χ*^2^(2) = 66.500, *p* < 0.0005. There was a statistically significant difference between all scan modalities (3Ds and 3Dh (*Z* = −3.166, *p* = 0.002); 3Ds and iCT (*Z* = −5.254, *p* < 0.0005) and 3Dh and iCT (*Z* = −6.002, *p* < 0.0005)) (see Fig. 3).

### Articular step-off

There was no statistically significant difference in the proportion of correctly evaluated step-offs, *χ*^2^(2) = 2.000, *p* = 0.368. No statistically significant difference for measurements of step-off depth [mm] between imaging modalities was shown: *F*(2.000, 98.000) = 0.580, *p* = 0.562, partial *η²* = 0.012.

**Figure 4:**
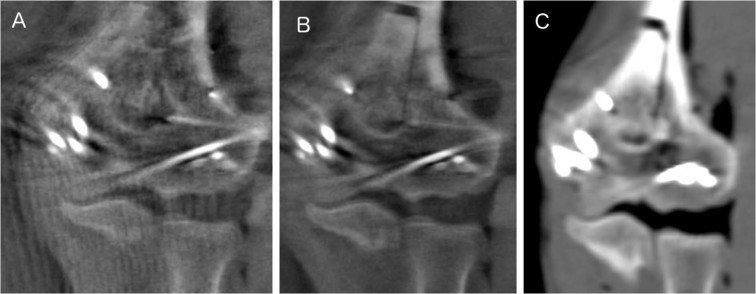
Step-offs in different imaging modalities (**A**: 3Ds, **B**: 3Dh, **C**: iCT).

**Figure 5:**
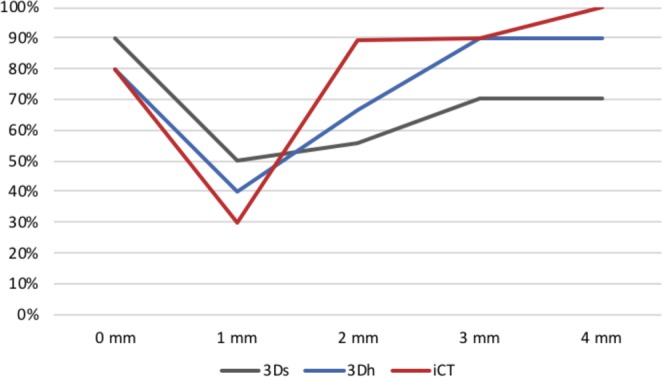
Percentage of correctly evaluated step-offs in relation to true step-off depth.

Only 30% of 1mm-step-offs were recognized in iCT, while 80% of 0 mm step-offs, 89% for 2 mm, 90% for 3 mm and 100% for 4 mm step-offs were correctly evaluated. Similarly, 40% of 1mm-step-offs were recognized in 3D, while 90%/80% of 0 mm step-offs, 56%/67% for 2 mm, 70%/90% for 3 mm and 70%/90% for 4 mm step-offs were correctly evaluted in 3Ds/3Dh (see Figs. 4 and 5).

### Articular impressions

**Figure 6:**
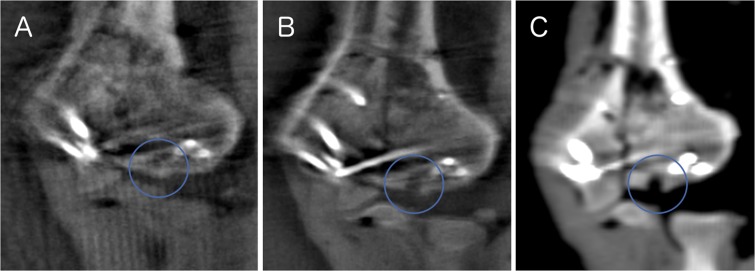
Impression in different imaging modalities (**A**: 3Ds, **B**: 3Dh, **C**: iCT).

For detecting articular impressions, the iCT was superior to the fluoroscopic imaging scans (see Fig. 6). iCT was more sensitive for articular impressions than 3D (3Ds: 40% accuracy (20/50), 3Dh: 40% (20/50), iCT: 70% (35/50)). There was a statistically significant difference in the proportion of correctly evaluated images, *χ*^2^(2) = 18.000, *p* < 0.0005. A statistically significant difference between 3Ds and iCT as well as 3Dh and iCT, *p* = 0.003 was proved. No difference between 3Ds und 3Dh, *p* = 1.000. There was a significant difference for evaluation of the depth of the impression between iCT and the fluoroscopic imaging scans: mean performance levels showed a statistically significant difference between measurements, *F*(1.256, 61.535) = 21.594, *p* < 0.0005, partial *η²* = 0.306. Post-hoc analysis revealed a significant difference (*p* < 0.0005) in performance of 3Ds and iCT (−1.300, 95%-*CI*[−1.946;−0.654]) as well as in performance of 3Dh and iCT (−1.340, 95%-*CI*[−2.043;−0.637]). No significant difference (*p* = 1.000) for performance of the 3Ds and the 3Dh (0.040, 95%-*CI*[−0.243;0.323]).

### Intra-articular screw placement

No statistically significant difference in the proportion of correctly evaluated intraarticular screw courses (*χ*^2^(2) = 6.000, *p* = 0.050, 3Ds and iCT as well as 3Dh and iCT, *p* = 0.250, 3Ds und 3Dh, *p* = 1.000) was shown. The average largest visible intra-articular screw course [mm] was 2.72 mm in 3Ds, 3.00 mm in 3Dh and 3.10 mm in iCT. No statistically significant difference for measurements of maximal length of intraarticular screw placement [mm] between imaging modalities (F(2.98) = 4.006, p = 0.021, partial η² = 0.076, 3Ds and iCT *p* = 0.056 (−0.380, 95%-*CI*[−0.767;0.007]), 3Ds and 3Dh *p* = 0.065 (−0.380, 95%-*CI*[−0.572;0.012]), 3Dh and iCT *p* = 1.000 (−0.100, 95%-*CI*[−0.499;0.249])).

A summary of the results is shown in Table 3.

**Table 3:** ^a^Friedman-Test, ^b^Cochran’s Q test, ^c^repeated measures ANOVA.

	3Ds	3Dh	iCT	*p*
Assessability score	0.60	0.96	1.80	<0.0005^a^
**Step-off**				
Correct evaluation	68%	74%	78%	0.368^b^
Mean max. measured expression of step-off [mm]	1.04 ± 1.14	1.16 ± 1.11	1.14 ± 1.09	0. 562^c^
**Articular impressions**				
Correct evaluation	40%	40%	70%	<0 0.0005^b^
Mean max. measured Impression depth [mm]	0.72 ± 1.20	0.68 ± 1.20	2.02 ± 1.97	<0 0.0005^c^
**Intraarticular screw placement**				
Correct evaluation	84%	84%	86%	0.050^b^
Mean max. measured intraarticular screw course [mm]	2.72 ± 1.49	3.00 ± 1.64	3.10 ± 1.82	<0.021^c^

### Interrater agreement

The interobserver agreement was low for assessability score (kappa = 0.137), fair for assessment of articular step-off (kappa = 0.349) and articular impression (kappa = 0.284). For intraarticular screw detection interrater agreement was moderate (kappa = 0.475).

## Discussion

The anatomical reconstruction of joint fractures is one of the decisive factors in order to achieve a good functional result^7^. Intraoperative detection of insufficient fracture reduction or implant placement allows immediate correction of the malposition and can thus prevent revision operations and improves patient outcome. Intraoperative fluoroscopy has become the standard method for assessing reconstruction of joint surfaces^14,15^. Still 2D-imaging often is not sufficient for the evaluation of fracture reduction and implant placement particularly in complex anatomical regions. In regard to these restrictions, the additional use of intraoperative 3D-imaging over biplanar fluoroscopy have been analyzed in several studies and been shown to reveal findings that are not visible in 2D-imaging^16,22,23^. For that reason, biplanar fluoroscopy was not included in this study as the intention was to compare two technical different modalities of intraoperative 3D-imaging. 3D-imaging delivers significantly better diagnostic results than two-dimensional images - especially in the cases of complex fractures and poor assessability of the surgical field. The limited field of view (often a cube with an edge length of 12–14 cm) and the presence of artifacts caused by implants in the radiation path can considerably reduce the assessability of the images^28^. Comparably, intraoperative CT-imaging offers a higher imaging quality and bigger field of view^26^ and less generation of artifacts. Intraoperative 3D-imaging is an essential key for controlling the surgical outcome of articular fractures. In order to ensure the highest possible accuracy of the assessment of the fracture site and thus the best clinical results, optimal intraoperative imaging is essential. iCT imaging offers the highest assessability for the surgeon. The four aspects in image assessment (assessability, impressions, step-offs and gaps) were evaluated independently. Thus, superiority was rated separately for each of these aspects. For detecting articular impressions, the iCT was superior to the fluoroscopic imaging scans. However, no statistically significant difference in the proportion of correctly evaluated step-offs or screw-placements was shown. Image quality with intraoperative CT is higher and certain factors of the reconstruction of joint surfaces can be better evaluated. Particularly noteworthy is the detection of articular impressions: impressions are an essential factor of fracture morphology in many joint fractures and are important for the functional outcome of the extremity. Yet articular impressions are particularly difficult to assess with implants in place often causing artifacts in 3D fluoroscopy. For avoiding false evaluation of articular morphology iCT is helpful for detecting articular impressions in presence of potentially artifact generating implants.

The difference in correctly evaluated step-offs for 1 mm steps in comparison to the larger step-offs can be explained with the voxel size. Voxel size for Arcadis Orbic (3D) is 0.47 × 0.47 × 0.47 mm³ compared to 1 × 0.75 × 0.75 mm^3^ ^29^ for intraoperative CT Airo which explains the poor recognition of 1 mm steps especially for iCT and can certainly be regarded as a disadvantage of intraoperative 3D imaging with iCT.

In general, an insufficient anatomical reduction (i.e. articular step-offs and gaps) will produce a poor clinical outcome. However, settings below one millimeter in step-off are not only extremely difficult to record but also particularly difficult to correct - therefore such findings could be regarded as sufficient clinical alignment.

The interrater agreement was rather low, showing low to moderate accordance. Possible explanations are the different levels of expertise of the raters, while all of them had experience in assessing 3D imaging data sets. Another reason might be that this kind of imaging intrinsically underlies a high degree of subjectivity what is reflected in the low kappa values. This underlines the need for standardized assessment guidelines and training of the surgeons that use this imaging in their daily routine.

Regarding limitations of the study, the difficult blinding of the observers to the modality has to be mentioned: for an experienced observer the differences between fluoroscopic imaging and CT scans are rather obvious due to different interfaces of the scan modalities and the apparent difference in imaging quality. Another aspect that can be addressed is the use of three osteosynthesis plates. Double plating of the distal humerus with two plates perpendicular to each other as well as osteosynthesis of the olecranon poses an extensive amount of potential artifacts. This is certainly a setting with a maximum of inserted osteosynthesis material - although not an unrealistic situation. Statistical analysis could have been improved if a comparison towards the gold standard – postoperative stationary CT – had been performed. Due to study conditions, the study design was created to compare intraoperative modalities.

Intraoperative 3D-imaging has particularly high diagnostic advantages in complex surgical procedures or poor assessability of the surgical field. iCT delivers even higher levels of image quality and accurate depiction of articular impressions than conventional 3D-imaging.

## Conclusion

Intraoperative CT imaging is more precise than 3D fluoroscopic imaging for detection of articular impressions. Articular step-offs and intraarticular screw placement are similar for iCT and 3D. Subjective imaging quality is the higher for iCT than for 3D fluoroscopic imaging. Intraoperative CT may therefore be particularly useful in assessing articular impressions and providing a good subjective image quality for the surgeon.
